# Case Report: Pancreatic Paraganglioma Mimicking a Well‐Differentiated Neuroendocrine Tumor: The Importance of Cytokeratin Immunohistochemistry

**DOI:** 10.1002/dc.70171

**Published:** 2026-07-01

**Authors:** Tanner Storozuk, Marc Vecchio

**Affiliations:** ^1^ University of Vermont Health Burlington Vermont USA

**Keywords:** pancreas, pancreatic paraganglioma, paragangliomaneuroendocrine

## Abstract

Paragangliomas (PGLs) are uncommon neuroendocrine tumors most commonly found in the head and neck that originate from neural crest cells. Primary pancreatic paraganglioma is exceedingly rare and has largely been described in case reports and small case series. We present the case of a 79‐year‐old woman with an incidental pancreatic head lesion initially favored to represent a well‐differentiated neuroendocrine tumor. Endoscopic ultrasound with rapid on‐site cytopathologic evaluation demonstrated neoplastic cells with neuroendocrine features. Further immunohistochemical (IHC) workup demonstrated tumor cells negative for cytokeratins and positive for GATA‐3, supporting a diagnosis of paraganglioma over a well‐differentiated neuroendocrine tumor. Cytokeratin immunohistochemistry should be employed alongside neuroendocrine markers in the workup of neuroendocrine tumors to avoid misdiagnosis.

## 1 Introduction

Paragangliomas (PGLs) are uncommon neuroendocrine tumors that arise from neural crest cells and can occur in both the sympathetic and parasympathetic nervous systems. Within the adrenal gland, these tumors are known as pheochromocytomas (PCCs). Collectively, these tumors are termed pheochromocytoma/paraganglioma (PPGL). Although all PPGLs possess metastatic potential, reliable histologic criteria for predicting behavior remain limited [1, 2, 3]. Epidemiologically, the estimated incidence is 2–8 cases per million people. PGLs are reported slightly more frequently in women, with female prevalence ranging from 51% to 57%. The median age of diagnosis ranges between 48 and 55 years [4]. Patients with PPGLs may present with symptoms of catecholamine excess, including hypertension, headaches, diaphoresis, and palpitations. However, many cases are completely asymptomatic (as was in the present case). Slightly over 50 cases of PGLs presenting as pancreatic masses have been reported in the past 50 years [3]. We present a case of a pancreatic paraganglioma, contributing to the limited literature describing this uncommon anatomic presentation.

## 2 Case Presentation

A 79‐year‐old female with a past medical history of hypertension, overactive bladder, and irritable bowel syndrome presented initially after a mechanical fall from standing at home. The patient had a longstanding history of irritable bowel syndrome‐diarrhea managed with scheduled colestipol and as needed loperamide. The patient did not have any other gastrointestinal symptoms or previous oncologic history.

**FIGURE 1:**
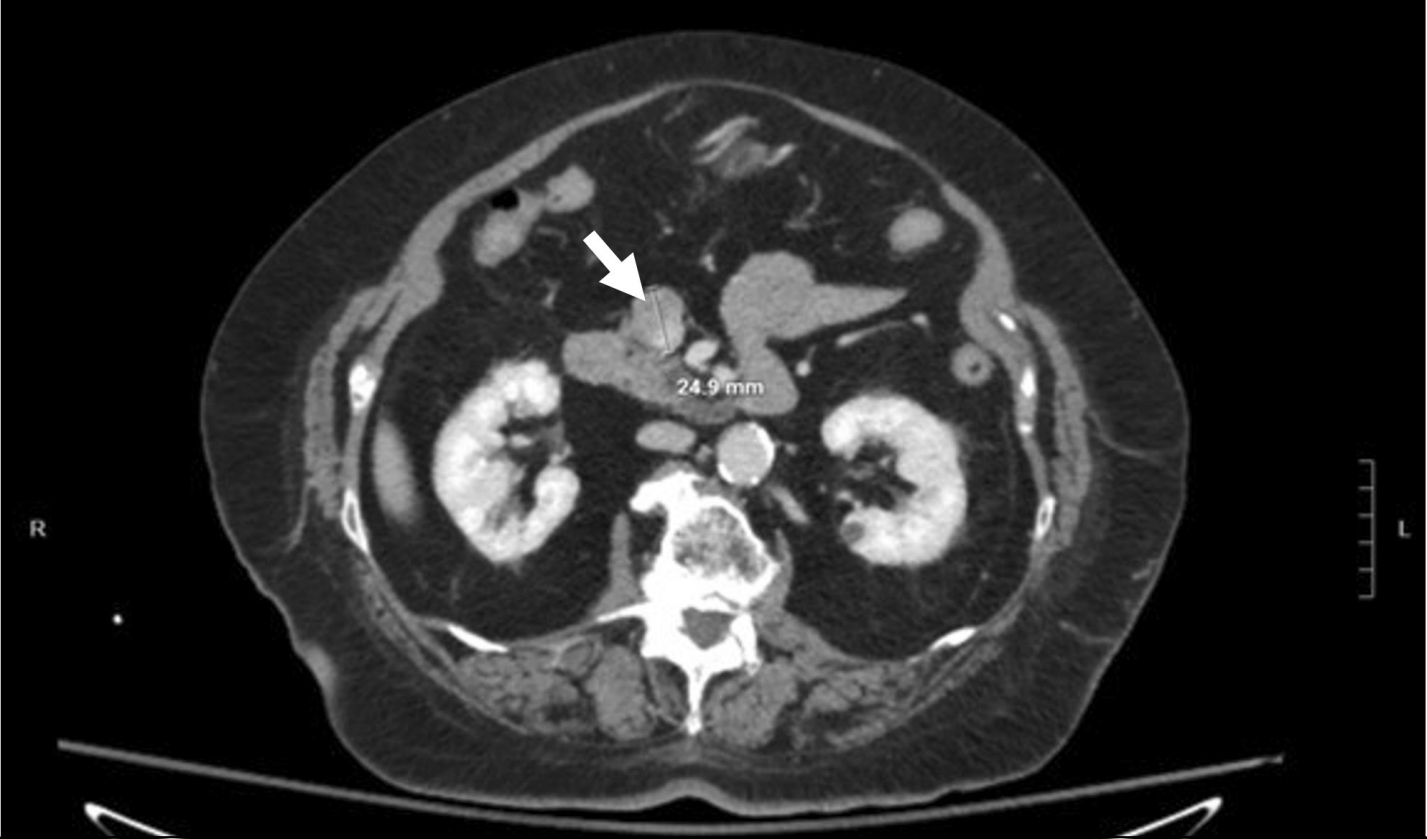
Computed tomography scan abdomen pelvis demonstrating a ~2.5 cm exophytic lesion arising from the pancreatic head (arrow).

Computed tomography scan of the abdomen revealed a 2.5 cm heterogeneously hyperattenuating exophytic lesion arising from the pancreatic head without dilation of the main pancreatic duct. Radiographically, the lesion was described as suggestive of a neuroendocrine tumor (Figure 1). A gastrointestinal consultation was requested for endoscopic ultrasound and fine needle aspiration.

**FIGURE 2:**
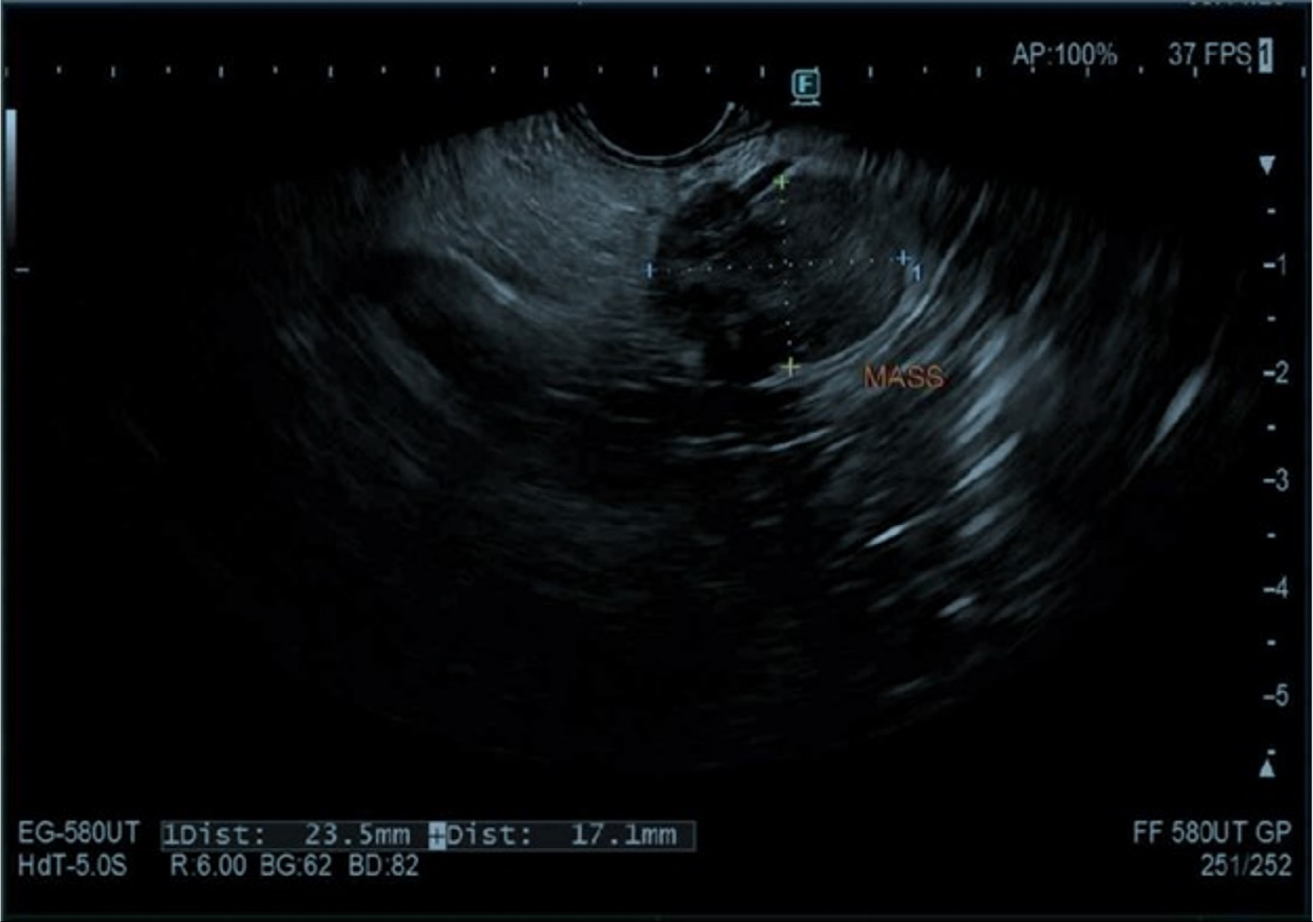
Endoscopic ultrasound of index pancreatic lesion. Hypoechoic mass of the pancreatic head measuring 23.5 mm × 17.1 mm.

**FIGURE 3:**
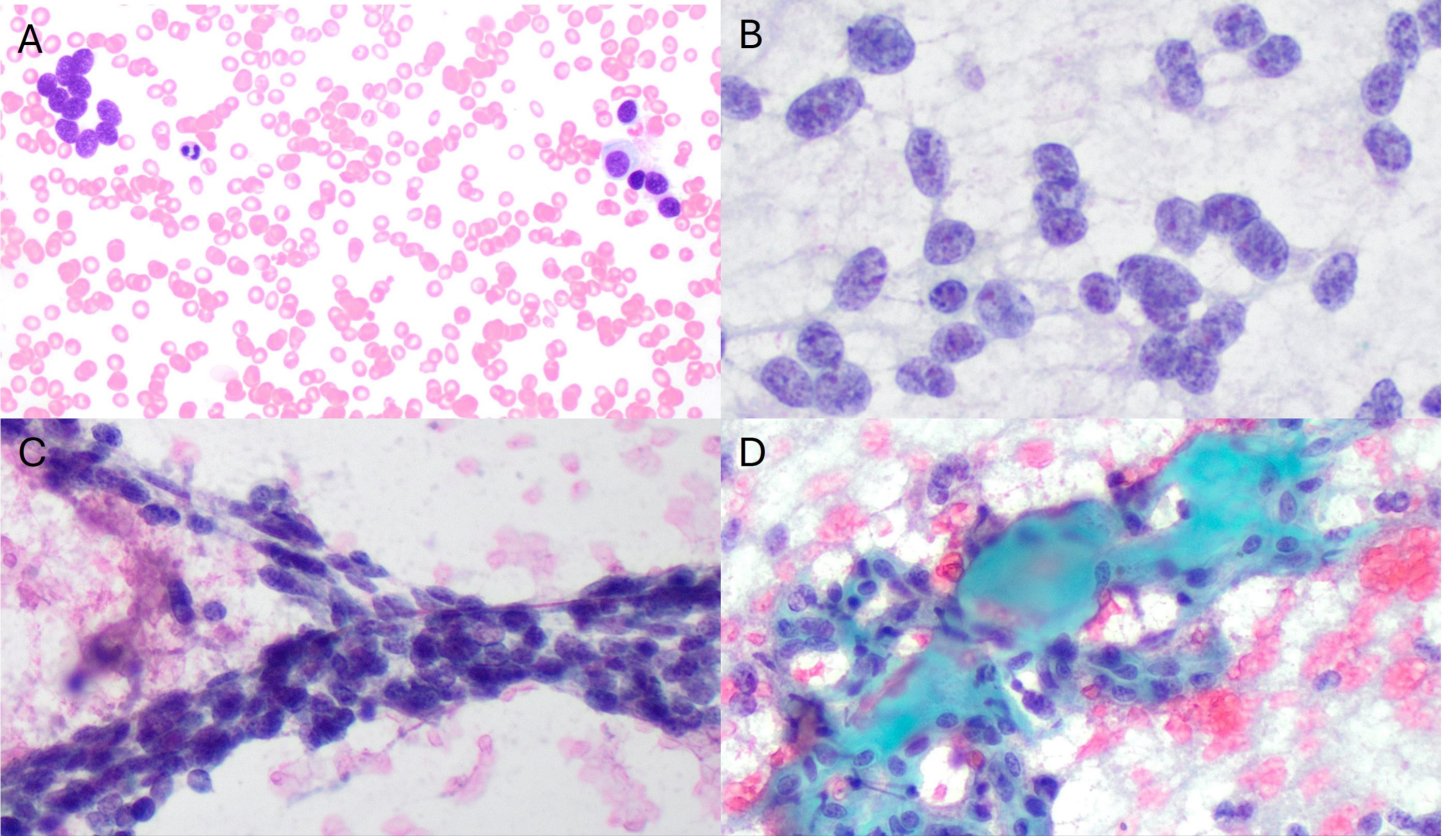
Cytology fine needle aspirates demonstrate loosely cohesive clusters of neoplastic cells with scant cytoplasm, round to oval nuclei, and speckled chromatin. (A) Air dried preparation stained with May Grunwald‐Giemsa (200×). (B) Alcohol fixed Papanicolaou stain (400×). (C) Alcohol fixed Papanicolaou stain demonstrating transgressing vessels (200×). (D) Alcohol fixed Papanicolaou stain with hyaline stroma (200×).

**FIGURE 4:**
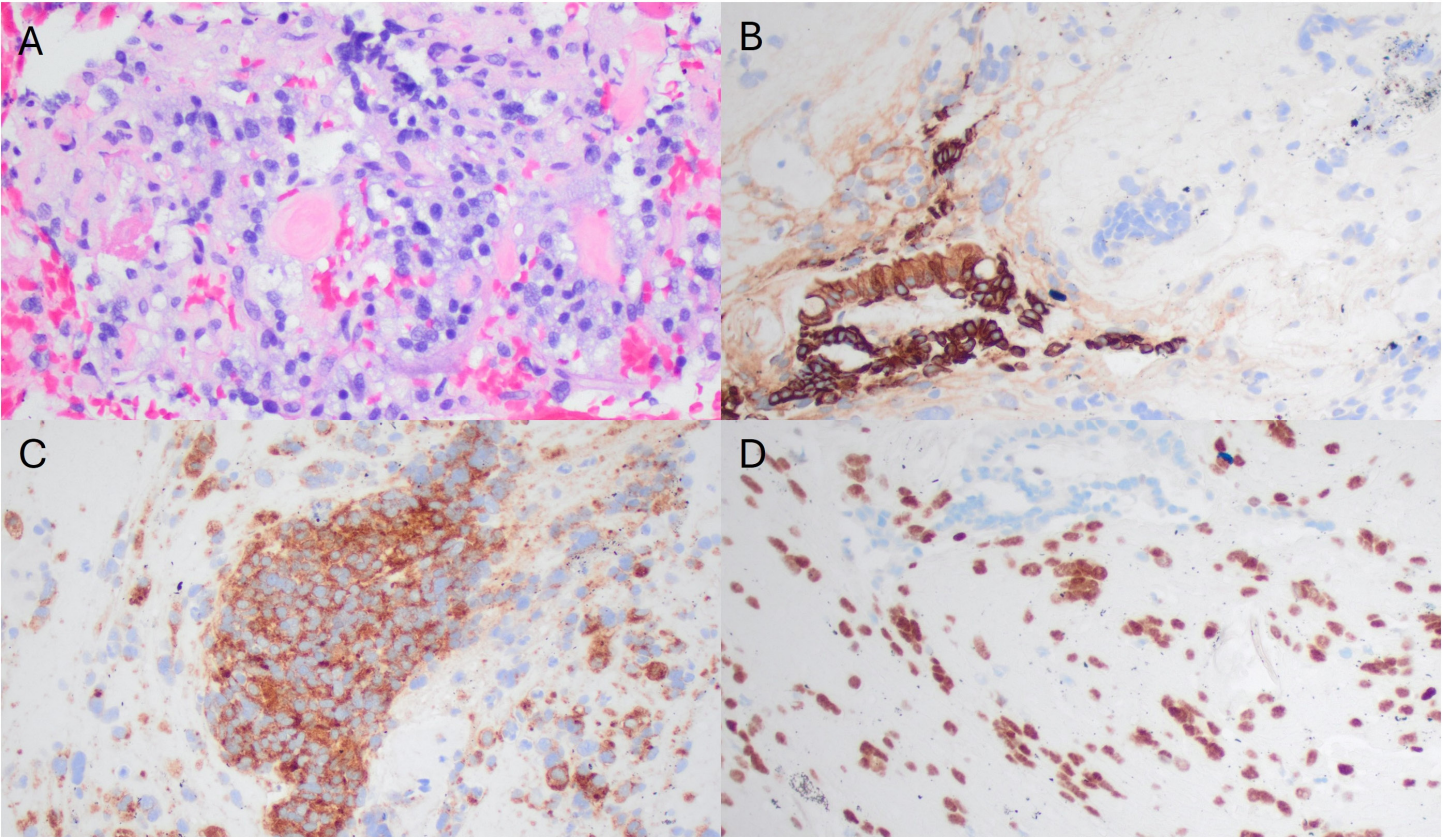
Cell block preparation showing neoplastic cells with cytologic features similar to aspirate smears. (A) Hematoxylin and eosin stain on cell block (200×). (B) Tumor cells are negative for cytokeratin AE1/AE3 (the positive internal control of gastrointestinal procedural contaminant) (200×). (C) Tumor cells are positive for synaptophysin (200×). (D) Tumor cells are diffusely positive for GATA‐3 (200×).

Outpatient endoscopic evaluation of the pancreatic lesion was performed which demonstrated a well‐circumscribed 23.5 × 17.1 mm hypoechoic mass protruding from the pancreatic head (Figure 2). The main pancreatic duct was not dilated. Endoscopic ultrasound guided fine needle aspiration was performed with rapid on‐site evaluation by cytopathology. Cytologic examination demonstrated a neoplastic population of loosely cohesive cells with nuclear size variability, finely granular chromatin with a “salt and pepper” appearance, and scant cytoplasm. Focal transgressing vasculature was identified. Hyalinized stroma was also appreciated (Figure 3). Congo red staining was performed to exclude amyloid and was negative. Immunohistochemical (IHC) workup was performed on the cell block material. The tumor cells were strongly and diffusely positive for synaptophysin, chromogranin, and GATA‐3, but negative for cytokeratins CAM5.2 and AE1/AE3 (Figure 4). The Ki‐67 proliferative index was low (< 3%). The combined cytologic and IHC features were compatible with paraganglioma.

Biochemical analysis to determine the functional status of the tumor has not been performed; however, the patient has no clinical symptoms suggestive of catecholamine excess. Molecular testing for germline susceptibility mutations has not been performed but may be pursued if clinically indicated.

## 3 Discussion/Conclusion

PGLs presenting as pancreatic masses are exceedingly rare, with approximately 53 cases reported in the literature over the past five decades. A literature review performed by Zhao et al. features a table summarizing these reported cases of pancreatic PGLs [3]. Several of the reported cases of pancreatic PGLs carried initial cytologic diagnoses of pancreatic neuroendocrine tumors [5, 6, 7]. Of the 53 reported cases, 21 underwent diagnostic endoscopic ultrasound guided fine needle aspiration, and 11 of these cases (52%) were initially misdiagnosed as neuroendocrine tumors. Only 7 cases (33%) were correctly diagnosed as paraganglioma on preoperative fine needle aspiration. On cytology alone, diagnosis is particularly challenging at this anatomic site because well‐differentiated neuroendocrine tumors are far more common in the pancreas.

Furthermore, PGLs and well‐differentiated neuroendocrine tumors exhibit substantial cytomorphologic overlap. Cytologic features include loosely cohesive tumor cells with round to oval nuclei and fine “salt and pepper” chromatin. Cell block preparation is therefore particularly important for establishing the correct diagnosis in these cases. In this case, a cell block was performed, increasing the cellular yield of the tumor cells, which aided the diagnostic workup.

A major diagnostic pitfall is failure to include cytokeratin immunohistochemistry in the workup of pancreatic neuroendocrine neoplasms. Pathologists should routinely employ cytokeratins and neuroendocrine markers (such as synaptophysin, chromogranin, or INSM‐1) in the initial workup. Diffuse absence of cytokeratin expression is atypical for well‐differentiated pancreatic neuroendocrine tumors and should prompt confirmatory cytokeratin stains together with GATA‐3 immunohistochemistry. Additionally, PAX8 immunohistochemistry may be positive in pancreatic neuroendocrine tumors (reported in up to 67% of cases) and is typically negative in PGLs [8]. S‐100 immunohistochemistry may also highlight the sustentacular cells of paraganglioma. These stains may also be helpful in this differential diagnosis but were not performed in this case. Recognition of absent cytokeratin expression in conjunction with GATA‐3 positivity is essential to avoid misclassification of pancreatic paraganglioma as a well‐differentiated neuroendocrine tumor.

The distinction of paraganglioma from well‐differentiated neuroendocrine tumors has clinical ramifications. Functioning PGLs may be monitored through measurement of tumor‐secreted catecholamines. The catecholamine profile may be important to clinical management and monitoring the patient's disease. Second, more than 40% of patients with PGLs carry germline mutations involving one of many genes (including *RET, VHL, NF1, SDHB, SDHD, SDHC*). These mutations carry implications for disease progression, patient surveillance, and genetic counseling for family members [9].

Currently, no standardized treatment guidelines exist for pancreatic PGLs; however, surgery is the principal treatment modality. The choice of surgery would depend on the proximity of the tumor to pancreatic parenchyma and other neurovascular structures [3]. In this case, conservative management was favored because of the patient's functional status.

In summary, pancreatic paraganglioma is an exceptionally rare neoplasm that may closely mimic a pancreatic neuroendocrine tumor on imaging and cytology. Recognition of absent cytokeratin expression together with GATA‐3 positivity is a critical diagnostic clue. Routine incorporation of cytokeratin immunohistochemistry into the evaluation of pancreatic neuroendocrine neoplasms may help prevent misdiagnosis and facilitate appropriate clinical management, surveillance, and genetic risk assessment.

## Funding

The authors have nothing to report.

## Ethics Statement

Ethics approval was not required for this case report in accordance with institutional policy. The report was prepared in compliance with ethical standards and patient confidentiality was maintained throughout.

## Consent

Written informed consent was obtained from the patient for publication of this case report and any accompanying images.

## Conflicts of Interest

The authors declare no conflicts of interest.

## Data Availability

The data that support the findings of this study are available on request from the corresponding author. The data are not publicly available due to privacy or ethical restrictions.
